# Transplant Trial Watch

**DOI:** 10.3389/ti.2022.10838

**Published:** 2022-10-03

**Authors:** Simon R. Knight

**Affiliations:** ^1^ Oxford Transplant Centre, Churchill Hospital, Oxford, United Kingdom; ^2^ Centre for Evidence in Transplantation, Nuffield Department of Surgical Sciences, University of Oxford, Oxford, United Kingdom

**Keywords:** heart transplantation, rejection, surveillance, hypothermic oxygenated perfusion, liver transplantation, extended criteria donor

To keep the transplantation community informed about recently published level 1 evidence in organ transplantation ESOT and the Centre for Evidence in Transplantation have developed the Transplant Trial Watch. The Transplant Trial Watch is a monthly overview of 10 new randomised controlled trials (RCTs) and systematic reviews. This page of Transplant International offers commentaries on methodological issues and clinical implications on two articles of particular interest from the CET Transplant Trial Watch monthly selection. For all high quality evidence in solid organ transplantation, visit the Transplant Library: www.transplantlibrary.com.

**Randomised Controlled Trial 1**

Cardiovascular Magnetic Resonance for Rejection Surveillance After Cardiac Transplantation.

*by Anthony, C., et al. Circulation 2022; 145(25): 1811–1824.*

## Aims

The aim of this study was to investigate the feasibility of cardiovascular magnetic resonance (CMR)-based monitoring for cardiac allograft rejection.

## Interventions

Participants were randomised to receive either CMR-based or endomyocardial biopsy (EMB)-based rejection surveillance.

## Participants

40 orthotopic heart transplant recipients.

## Outcomes

The primary endpoint was frequency and cumulative freedom from significant (>grade 2R) rejection. The secondary endpoints included frequency and cumulative freedom from low-grade (grade 1R) rejection, kidney function, hospitalisation, duration of hospital stay, infection, myocardial function, death, immunosuppression exposure and the incidence of biopsy-related complications.

## Follow-Up

1 year.

## CET Conclusion

This is an interesting and well-conducted study in cardiac allotransplantation. Cardiac magnetic resonance (CMR) imaging was compared to the standard protocol of surveillance for rejection with the invasive endomyocardial biopsy (EMB). The first part of the study was a cross-sectional analysis to understand cut-off values for acute rejection and the second part was a randomised study comparing the two surveillance methods using this information. The trial was set up as a noninferiority study and therefore the inclusion did not need to be large (20 in each arm), as per prior power calculation. A detailed analysis of the CMR validation is provided. The primary endpoint was frequency of significant rejection (grade 2R or higher). This was found to be similar in the two groups. In order not to miss high grade rejection, treating physicians could request EMB on any patient at their discretion. This option was taken 11 times in the CMR group and in 9 of these cases the EMB result was identical to the CMR result. In this single-centre study, a surveillance protocol using CMR instead of EMB in cardiac allograft recipients was safe, feasible and offers significant advantages over invasive cardiac biopsies.

## Jadad Score

3.

## Data Analysis

Strict intention-to-treat analysis.

## Allocation Concealment

Yes.

## Trial Registration

ACTRN12618000672257.

## Funding Source

Non-industry funded.

**Randomised Controlled Trial 2**

Hypothermic Oxygenated Perfusion in Extended Criteria Donor Liver Transplantation—A Randomized Clinical Trial.

*by Ravaioli, M., et al. American Journal of Transplantation [Online ahead of print].*

## Aims

The aim of this study was to compare the effect of hypothermic oxygenated perfusion (HOPE) vs. static cold storage (SCS) in extended criteria donor (ECD) liver transplantation.

## Interventions

Participants undergoing transplantation of an ECD liver graft were randomly assigned to receive a liver after HOPE or after SCS alone.

## Participants

135 potential ECD liver grafts were randomised, of which 110 were used for liver transplantation.

## Outcomes

The primary outcome was the incidence of early allograft dysfunction (EAD). The secondary outcome were patient survival, graft survival, the early allograft failure simplified estimation (EASE) risk score, and the rate of graft or other graft-related complications.

## Follow-Up

1 year.

## CET Conclusion

This is an interesting and well-conducted trial in ECD liver transplantation. Livers were randomised to standard static cold storage (SCS) or to a period of Hypothermic Oxygenated Perfusion (HOPE), using the Vitasmart device (Bridge to Life, DG, United States). Organs in the HOPE group had a period of SCS of 4–5 h on average prior to starting HOPE for 2–3 h on average. No organ was discarded during perfusion. The study was single centre and designed with a prior power calculation to determine sample size. The primary endpoint was Early Allograft Dysfunction (EAD) using a well-established composite definition. There was a significant reduction in EAD with HOPE compared to SCS (13% vs. 35%) and also a significant reduction in re-transplantation (0% vs. 11%). This form of HOPE, using just portal vein perfusion in ECD liver transplantation, is associated with better early allograft function, which is very likely to impact on longer term function and graft survival.

## Jadad Score

2.

## Data Analysis

Per protocol analysis.

## Allocation Concealment

No.

## Trial Registration

ClinicalTrials.gov—NCT03837197.

## Funding Source

Non-industry funded.

## Clinical Impact Summary

The shortage of suitable donors to meet demand has resulted in increasing use of extended criteria donor (ECD) organs to try to address the mismatch. ECD donor organs are known to be more at risk of adverse post-operative outcomes due to increased vulnerability to ischaemia-reperfusion injury. In attempts to counter this additional risk, there has been a great deal of interest in novel perfusion technologies to recondition, repair and assess grafts prior to transplant. Such technologies can be used in the donor (normothermic regional perfusion, NRP) or *ex-vivo* (hypothermic oxygenated perfusion, HOPE or normothermic machine perfusion, NMP). The technologies differ in their simplicity/ease of use, ability to assess organ viability and the duration of safe perfusion.

In a recent paper in the American Journal of Transplantation, Ravaioli and others report a single centre randomised controlled trial of HOPE after static cold storage (SCS) versus SCS alone in ECD liver grafts (1). 110 recipients were randomised and followed for a median of 473 days. The authors report a significant reduction in the risk of the primary endpoint of early allograft dysfunction with HOPE, from 35% to 13%. This reduction is similar in magnitude to that seen in previous studies of NMP (2) and HOPE in DCD livers (3). Unlike in these previous studies there was no difference in incidence of biliary complications, most likely as this study does not include DCD livers which are at higher risk for ischaemic-type biliary lesions.

Perhaps the most striking finding is that graft survival was significantly higher in the HOPE arm of the study, a finding not seen in the larger multicentre studies of HOPE or NMP. A detailed breakdown of causes and timings of graft loss is not provided, making the role of perfusion in this finding difficult to interpret. Another interesting finding is the numerically lower incidence of acute rejection in HOPE livers. This has been seen previously with use of HOPE in kidney transplantation (4), and may offer at least a partial explanation for the difference in graft survival seen.

Overall, these findings support previous studies in both liver and kidney transplantation that HOPE is a safe, simple and effective method of preservation which may be beneficial in marginal donor organs.
